# Fast food fever: reviewing the impacts of the Western diet on immunity

**DOI:** 10.1186/1475-2891-13-61

**Published:** 2014-06-17

**Authors:** Ian A Myles

**Affiliations:** 1 Bacterial Pathogenesis Unit, Laboratory of Clinical Infectious Diseases, National Institute of Allergy and Infectious Diseases, National Institutes of Health, 9000 Rockville Pike Building 33, Room 2W10A, Bethesda, MD, 20892, Maryland

## Abstract

While numerous changes in human lifestyle constitute modern life, our diet has been gaining attention as a potential contributor to the increase in immune-mediated diseases. The Western diet is characterized by an over consumption and reduced variety of refined sugars, salt, and saturated fat. Herein our objective is to detail the mechanisms for the Western diet’s impact on immune function. The manuscript reviews the impacts and mechanisms of harm for our over-indulgence in sugar, salt, and fat, as well as the data outlining the impacts of artificial sweeteners, gluten, and genetically modified foods; attention is given to revealing where the literature on the immune impacts of macronutrients is limited to either animal or in vitro models versus where human trials exist. Detailed attention is given to the dietary impact on the gut microbiome and the mechanisms by which our poor dietary choices are encoded into our gut, our genes, and are passed to our offspring. While today’s modern diet may provide beneficial protection from micro- and macronutrient deficiencies, our over abundance of calories and the macronutrients that compose our diet may all lead to increased inflammation, reduced control of infection, increased rates of cancer, and increased risk for allergic and auto-inflammatory disease.

## Introduction

The Western diet is characterized by a high intake of saturated and omega-6 fatty acids, reduced omega-3 fat intake, an overuse of salt, and too much refined sugar [1]. Most are aware that this type of eating, if not in moderation, can damage the heart, kidneys, and waistlines; however, it is becoming increasingly clear that the modern diet also damages the immune system. The modern lifestyle is also typified by reduced exposure to microorganisms, increased exposure to pollutions, heightened levels of stress, and a host of other exceptionally well reviewed variables that likely contribute to immune dysfunction [2]. Therefore, while dietary effects on immunity should not be thought of in isolation, herein we focus on the body of evidence detailing the mechanisms for the Western diet’s impact on immune function.

### Total nutrient intake

Intake of adequate calories and micronutrients is vital for optimal immune function. Deficiency in total calories and/or protein, as seen in parts of the world stricken with starvation, severely reduces the immune system’s ability to respond [3]. As one example, inflammatory cytokines are themselves proteins, and thus infection during starvation can lead to the production of these cytokines at the expense of other proteins from blood and tissues [3]. However, the obesity epidemic clearly outlines that today’s diet contains an over abundance of nutrients [1]. While the Western world is not free from micronutrient deficiencies, since this review aims at detailing the immuno-nutrition of a Western diet not typically characterized by micronutrient deficiency, they are thus considered outside the focus. Therefore, we encourage interested readers to seek out lovely review articles on the immune impacts and mechanistic understandings of dietary minerals and vitamins [3-6].

Adipocytes release inflammatory substances including interleukin (IL-) 1, IL-6, and tumor necrosis factor (TNF) [7]. In animal models, it appears that these signals can act as false alarms that, over enough time and in large enough amounts, cause the entire system to dial down its responsiveness – analogous to a person removing a battery from a twitchy smoke detector that frequently alarmed when no signs of fire were present [7,8]. When an actual infection comes along, the response may be delayed because the early warning system was silenced – just as deactivating that smoke detector leaves a home more susceptible to fire [7]. While human verification is lacking, this concept is not unique to immunity, for example anabolic steroid abusers down regulate their steroid responses [9] while opioid abusers down regulate innate opiate responses [10].

Obese individuals have fewer white blood cells to fight infection and those cells they do possess have reduced phagocytosis capability [11,12]. While a complex interplay of hormonal, metabolic, and immunologic processes contribute to the biologic responses in the obese the resultant immune dysfunction increases the risk of infections of the gums, respiratory system, and of surgical sites after an operation [13-17]. Even routine interventions like immunizations may not work as well simply due to the inability of the vaccination needle to reach the muscle tissue of the arm [18]. One possible mechanism for obesity’s harmful effects on the immune system could be the increased levels of leptin in the blood. All mononuclear immune cells have a receptor for leptin and activation leads to an increase in IL-1, IL-6, and TNF [19]. Leptin stimulates NK cells, activates the transcription factor STAT3, and reduces the anti-inflammatory T-regulatory (Treg) cells [20]. In general, adiponectin has opposing effects on immunity and interestingly the ratio of the two can predict the development of coronary artery disease in diabetics [21]. Hypothetically, as with the development of resistance to leptin’s appetite suppressing functions [19], patients with obesity may, overtime, down-regulate the immune activation attributable to leptin [7]. However intriguing the in vitro impacts or correlation evidence of leptin may be, the precise mechanism by which excess calories impact the immune system has yet to be fully elucidated and will be very difficult to separate from the underlying mechanisms of the macronutrient sources of calories.

Eating disorders currently attributed to image obsession are also an unfortunate part of modern dietary habits and their immune impacts have gone relatively ignored compared to obesity. While the nutrient deficiencies seen in eating disorders are not nearly as severe as for the starvation seen in developing nations, subtle deficiencies appear to lead to subtle immune defects [22]. Both anorexia and bulimia may reduce neutrophil and monocytes numbers, T-cell number and function, anti-bacterial complement proteins, as well as effects attributable to any micronutrient deficiency that may develop [23]. Yet surprisingly, despite infection being a major cause of death in patients with anorexia, most are infection free until the late stages of their disease; speculation on the cause of this finding proposes that either the severe iron deficiency and sequestration render the blood a harsh environment for pathogen survival or that anorexic patients are, typically, deficient in carbohydrates and calories but may be only moderately deficient in proteins and fat [22,23]. Further investigation into the immune impacts of eating disorders is warranted.

### Sugars, salt, and fats

#### Sugar

In vitro evidence suggest processed, simple sugars also reduce white blood cell phagocytosis and possibly increase inflammatory cytokine markers in the blood [24,25]; of note, the author’s attribute their findings more to the relative glycemic load of meals than the sugars themselves and the most direct study on sugar’s effect on lymphocyte function is now four decades old and thus, repeat investigation employing in vivo and/or modern techniques is required. Meanwhile, the complex carbohydrate fiber (but not starches), such as that found in fruits and vegetable, appear to reduce inflammation in both humans [26-32] and mice [33]. The impacts of artificial sweeteners are less clear; provocative, yet highly limited, evidence implicates saccharin and sucralose as contributors to Crohn’s and Ulcerative Colitis via interference with homeostatic inactivation of digestive proteases [34,35]. However this evidence is only epidemiologic correlation and animal modeling, and lacks direct human investigation. Other studies looking at the effects of sweeteners in cell culture suggest anti-inflammatory effects in the blood [36,37]. Few studies on newer sweeteners have been conducted, yet limited cell-culture evidence on stevioside suggests anti-inflammatory properties while improving phagocytosis and mitogen responses for both T and B cells [38-40]. Potential immune impacts of the newest sweetener, mongroside V, have not been directly investigated. Therefore, definitive commentary on the immune impacts of sweeteners will require further investigation.

#### Salt

Animal studies suggest that high salt in the diet might also increase IL-17-mediated inflammation and could worsen autoimmune diseases, although predictions on how this may affect humans should only be seen as preliminary [41,42]. There is however ample evidence on how dietary fat affects the immune system.

#### Saturated fatty acids

One potentially harmful effect of fat is enhancement of the prostaglandin system as it feeds into the arachadonic and prostaglandin E2 (PGE_2_) pathways [43]. PGE_2_ is pro-inflammatory, increasing IL-17 production and macrophage activation among other pathways [43]. Additionally, dietary fats alter the lipids of the membranes of immune cells, disrupting the immune functions [44,45]. Yet perhaps the most concerning aspect of modern dietary fat is its ability to directly trigger the inflammatory process.

One of the first-line weapons the immune system deploys against infection are molecules called Toll-like receptors (TLR). While complex in its workings, when the immune system comes across a potential invader these receptors are designed to evaluate if it is bacterial, viral, or fungal. If the body finds evidence of any of these organisms, the immune system can begin its attack immediately while the adaptive immune system assesses what specific pathogen it is facing [46]. One of the TLR weapons, TLR4, is designed to sense bacteria. Unfortunately the part of the bacteria TLR4 binds, lipopolysaccharide (LPS), contains mostly saturated palmitic and steric fatty acids [47-51]. Meaning that TLR4 can generate inappropriate signaling when exposed to certain saturated fats if in too great of frequency, amount, or homogeneity rather than in a more biological balance and dosage. Any resultant, abnormal signaling may lead to a misguided attack upon saturated fat when it is perceived as a bacterial invader [9,47-54]. The resulting inflammation in the gut can lead to a break down of barriers, allowing harmful substance to leak from the gut into the blood stream and contribute to immune dysfunction that worsens infection control [52,54,55]. Consistent with these in vitro and animal models, studies in humans reveal down regulation of TLR4 and increased LPS translocation occurring within hours of a bolus of saturated fat [54,56], while polymorphisms reducing TLR4 functioning are relatively protective against dyslipidemia, coronary artery disease, and metabolic syndrome [57,58]. Saturated fats interact with another bacterial receptor, TLR2 and its co-receptor CD14 (which is shared with TLR4), and thus may impact infectious outcomes for both Gram-negative infections such as *E. coli* as well as Gram-positive infections like *Staph. aureus* and have even be implicated in coronary artery disease pathogenesis [49,52,59,60].

#### Omega-6 fatty acids

While saturated fats are the most inflammatory [50,61,62], overabundance of omega-6 (n-6) poly-unsaturated fats, such as those found in most cooking oils, have also been implicated in immune response through several mechanisms including effects on TLR4 [53] and serving as precursors for inflammatory mediators [63-67]. However, a recent review of human trials seems to undermine evidence supporting n-6 intake increases inflammation [68]. The meta-analysis found the only measured mediator that was significantly altered by an increase in dietary linoleic acid was PGE_2_ and concluded that the lack of detectable impact in human clinical trials indicates that the cell-culture and animal based evidence against n-6 fails to accurately reflect the complexity of human physiology. However, one confounder shared by many of the clinical trials investigating the immune impacts of fatty acids may be their exclusion of unhealthy subjects and subsequent lack of disease context. While retrospective studies are limited in their own ways, an intervention study investigating an ubiquitous exposure like dietary n-6 that excludes everyone with pre-existing inflammation may be excluding the biologically susceptible portion of the population and thus may be predestined to find no impact of their intervention; while the lack of baseline inflammation in clinical trails could alleviate the concern for omega-6 as a universal inducer of chronic inflammation, since these trials occur in the absence of either infectious challenge or underlying inflammatory disorders they make no comment on potential for n-6 intake to impact the risk for, control of, or inflammation during, acute pathology. Additionally, clinical trials involving n-6 have yet to measure inflammatory markers that are downstream of the elevated PGE_2_ (such as IL-17 and the Th17 pathway) [43] or directly investigate the TLR4 axis, and instead focus on the more traditional Th1 markers of TNF, C-reactive protein (CRP), or cardiovascular disease [69]. Furthermore, inflammation limited to the small bowel may not elevate typical blood inflammatory markers that are measured in these clinical trials despite imparting pathology [70]. Thus, further investigation into the immune impacts of omega-6 will be needed before any definitive connection between the provocative in vitro findings and human disease pathology can be stated.

#### Omega-3 fatty acids

The immune impact of trans unsaturated fatty acids (trans fats) have gone under investigated whilst researchers focus on their deleterious cardiovascular effects, however one study found an increase in IL-6 and CRP but only in the overweight female subgrouping [71]. In contrast, omega-3 (n-3) poly-unsaturated fats are generally associated with anti-inflammatory effects [63,72-74]. Dietary n-3 may have beneficial effects on a variety of conditions with inflammatory components, such as atherosclerosis and cardiovascular disease [73], inflammatory bowel [63], and allergic diseases [75,76]. Maternal intake of n-3 during pregnancy protects against the development of allergic and inflammatory disease in infants and children whereas diets rich in saturated and/or n-6 fats were associated with an increased risk [75,77]. Furthermore, n-3 directly interacts with transcription factors such as NFκB and PPAR-γ to down-regulate the expression of pro-inflammatory genes [63,73] and inhibits activation of TLR4 [48]. Omega-3 may further regulate the immune response through resolvins and protectins, anti-inflammatory mediators synthesized from eicosapentaenoic acid (EPA) and docosahexaenoic acid (DHA) [72,78-80]. These mediators reduce inflammation-induced neutrophil infiltration, promote the scavenging of inflammatory chemokines [79], and enhance macrophage phagocytosis to clear apoptotic cells [72]. Animal models also suggest omega-3 serves as an anti-inflammatory balance to modulate TLR2- and TLR4-dependent inflammation [45,81,82]. Thus, another potential contributor to modern diet-induced immune dysfunction may be the increased consumption of omega-6 in lieu of omega-3 fatty acids [1].

### Gluten

Recent animal and cell-culture models have found that elements in gluten can stimulate inflammation through TLR4 [83]; while these findings are afar from conclusive and require human correlation, they do conjure intriguing speculation given the current gluten-free dietary trend. This mechanism, even if eventually validated in humans, is in contrast with the reported primary mechanism of celiac disease; in celiac sprue, people with a very specific genetic pre-disposition have an error in gluten processing that leads to a different and more severe kind of immune activation. Dietary gluten is modified by tissue trans-glutaminases and, normally, proteins are digested by antigen presenting cells (APC) such as dendritic cells or B-cells; the proteins are then presented on a major histocompatibility complex (MHC) to T-cells for evaluation and, if deemed foreign, induce immune activation. In patients with celiac, a particular MHC receptor, either HLA-DQ2 or HLA-DQ8, allows the processed gluten to act as a sort of super-antigen, binding the APC to the CD4+ T-cell without going through normal processing; this inappropriately activates the T-cell and results in inflammation and symptoms [84-87]. However, HLA-DQ2 and HLA-DQ8 are found in approximately 40% of the US population and yet only roughly 1% of the US carries the diagnosis of celiac [88,89]. Therefore, some additional undiscovered mechanisms as well as genetic and/or environmental risk factors must be present.

### The microbiome and inheritance

**Figure 1:**
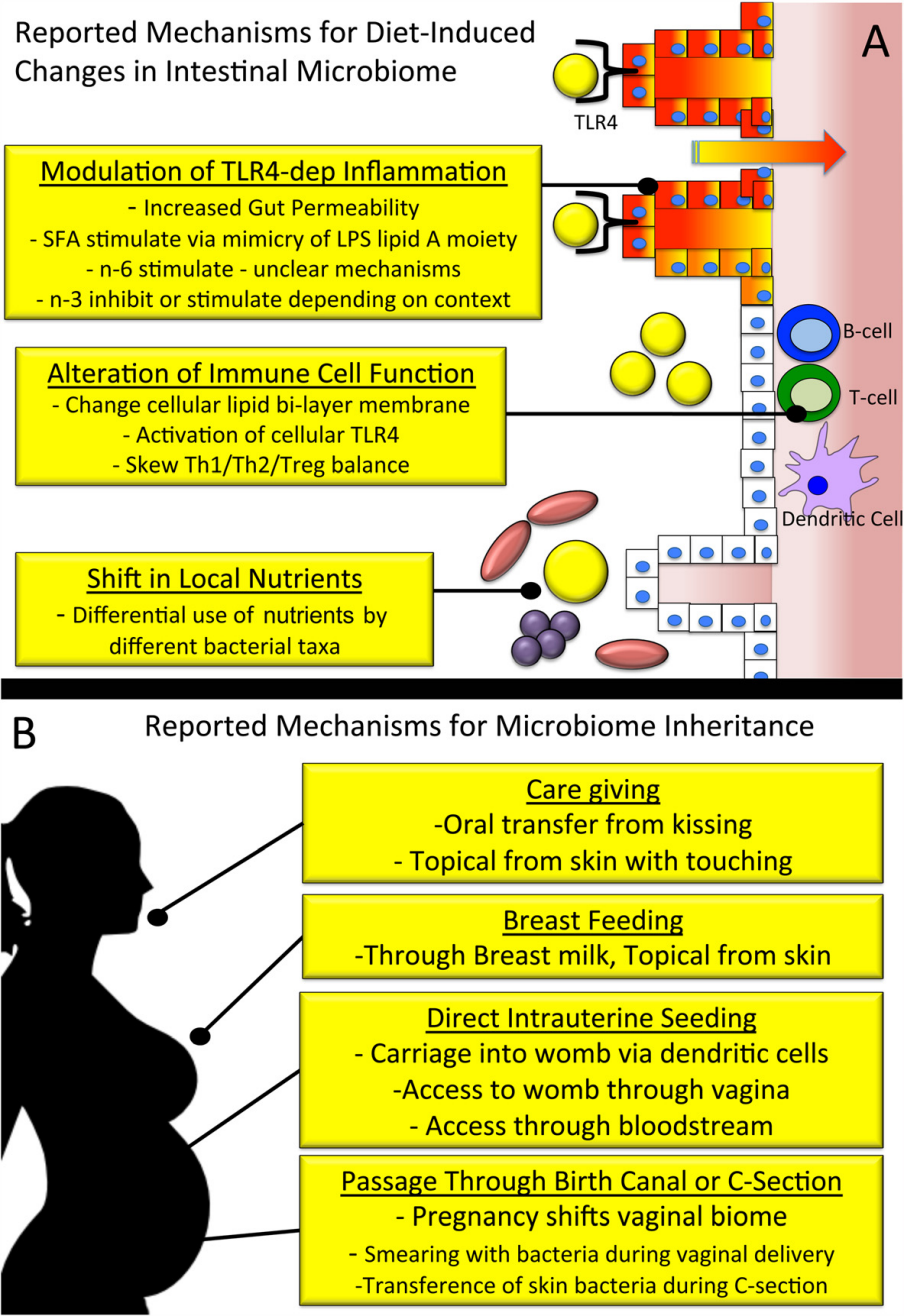
**Diagrammatic overview of select mechanisms for diet-induced inflammation, dysbiosis, and inheritance. (A)** Diagrammatic representation of the reported mechanisms for how diet alters the gut microbiome. **(B)** Summary of the current reported mechanisms for inheritance of the microbiome from mother to child. TLR4-dep, Toll-like receptor 4 dependent; SFA, saturated fatty acids; LPS, lipopolysaccharide; n-6, omega-6 fatty acids; n-3, omega-3 fatty acids; Th, T-helper cell; Treg, T-regulatory cell.

The notion that diet, stress, and environment can, for better or worse, imprint upon the bowel has been around since the ancient Egyptian pharaohs [90]. However, only recent focus and technologic advances have allowed accurate elucidation of the mechanisms by which our lifestyle impacts our microbiome and leads to dysbiosis. In the gut (and on the skin), there is an optimal, albeit not yet fully elucidated, balance of bacterial species. Some strains of bacteria are needed to digest dietary fibers [91] while others produce valuable nutrients like vitamin K [92]. Beneficial bacteria aide their hosts by occupying space and/or modifying the microenvironment in ways that prevent harmful bacteria from gaining a foothold [91]. More importantly, the commensal flora provides a type of training to the immune system. Like a sparing partner in boxing, the immune system’s interactions with the normal commensal flora provides an education that is indispensable when a pathogenic opponent is encountered. The current understanding on how dietary fats alter the microbiome include TLR4-dependent induction of local inflammation leading to altered host environment, shifts in immune cell membrane functions, and changes in nutrient availability favoring some organisms over others [47,52,93] (Figure 1A). Dietary simple sugars appear to lead to dysbiosis directly through changes in local nutrient concentrations and bacterial functions that may favor harmful taxa over the beneficial commensals [92,94-103]. Interesting preliminary culture-based and animal research has shown the gut microbiome to possess the ability to metabolize the artificial sweeteners considered otherwise non-caloric for humans. While results must be interpreted cautiously, gut bacteria can process sweeteners into various short-chain fatty acids (SCFA) that hold a wide array of potential consequences [104]; while some SCFA may be beneficial, their production may shift the bacterial balance [105,106], may be processed into absorbable byproducts that provide calories, and interestingly may activate the TLR4 pathway [104,107].

What is perhaps of larger concern is that the harmful effects of diet can actually stretch across generations. A mother’s diet may potentially shape her child’s flavor preferences even before birth, potentially skewing their palette towards anything from vegetables to sugary sweets in ways that could influence subsequent propensity for obesity and/or unhealthy dieting [108]. In addition, children inherit their microbiome from their mother mostly through parturition but also during breast-feeding and development until the bacterial balance matures around two to four years of age [92,109]. However, recent evidence suggests that the microbiome may also be seeded into the unborn fetus while still in the womb [109,110] (Figure 1B). When the mother’s diet causes a harmful imbalance of her bacteria, she passes this imbalance on to her child and thus fails to present the ideal commensals for a proper immune education during her child’s most critical developmental window [52]. This developmental dysbiosis leaves the offspring’s immune system poorly trained to fight off infections and encourages autoimmune and allergic diseases [52]. In mice, high dietary saturated fat solely during lactation led to a pro-inflammatory milk via a TLR2/4-dependent but microbiome-independent manner, furthering that saturated fat has additional direct harm to the newborn beyond the indirect harm steaming from dysbiosis [111]. Just as loss of honeybees from orchards or addition of an invasive species to a lake creates significant harm for the surrounding biosphere, so too it appears that small shifts in our microbiome caused by today’s unhealthy diets can reverberate through human health.

However, lest fathers believe that their diet does not impact the offspring, paternal epigenetics related to methylation of DNA and histones can also be inherited by the offspring and could alter early development of the immune system [52]. Epigenetic changes in DNA are, in effect, cellular memory; these changes prevent dividing pancreas cells from becoming cells of the kidney or any other organ [112]. This memory is so essential that many neoplasms begin with a cell’s loss of the epigenetic memory learned during embryogenesis [113,114]. Since the information encoded upon DNA is passed from parent-to-child and even potentially from parent-to-grandchild, cells that learn bad habits like ignoring signs of infection or over-reacting to antigens could combine with microbiome shifts to further worsen a child’s immunologic development [112,115,116]. The degree to which shifts in the microbiome can affect epigenetic changes in DNA, and vice versa, is currently not fully understood.

In addition to altering TLR-mediated inflammation and potentially DNA epigenetics, a mechanism by which alteration in microflora may drive immune-mediated disease involves the gut bacteria’s effect on regulatory T-cells (Tregs), the cell tasked with keeping the immune system in balance during both inflammation and homeostasis [117]. Alterations in the microbiome have been shown in both mice and (to a less extensive degree) humans to affect Treg development [118-122], and reduction in Treg signal is associated with worse outcomes in infection control [123], autoimmunity [124,125], allergic sensitization [126], and has been, more controversially, associated with cancer risks [127-129]. Recent mouse work has also shown transplanting the gut flora from allergic mice to wild type mice can significantly alter oral allergic sensitization [130], indicating direct effects of the gut flora on immune disease. Therefore, dietary choices that alter gut microbiome likely alter systemic responses through changes in the number and function of regulatory T cells.

Unraveling which specific bacterial strains are either the protectors or pathogens has not yet been elucidated in either mice or humans, however the field of microbiota research has many informative discoveries. The desire to foster a healthy microbiome is the driving force behind the therapeutic use of probiotics. Some studies have shown a positive impact for probiotic use including numerous and well reviewed immune beneficial effects of *Lactobacillus species* including alleviating traveler’s diarrhea, reducing respiratory infections, and serving as adjunct treatment for allergic rhinitis, asthma, and atopic dermatitis [116]. Supplementation with various *Lactobacillus, Lactococcus,* and *Bifidobacterium species* reduce the rate and severity of childhood atopic dermatitis when fed to pregnant women during the later weeks of gestation [131]. Additional studies have made use of transgenic bacteria producing the anti-inflammatory cytokine IL-10 to aide Crohn’s treatment [132] and even found relief of anxiety and depression through bacteria that naturally produce serotonin [133,134]. Evidence outlining associations between natural shifts in the microbiome and impacts on human health is also plentiful. In general, increased numbers Firmicutes relative to Bacteroidetes is associated with an increased incidence of allergy, asthma, and obesity [135]. As stated, processed sugars and saturated fats encourage dysbiosis [52,92] while complex carbohydrates encourage an anti-inflammatory microbiome and discourage growth of infections from *Clostridium difficile*[92]. Intake of omega-3 increases ratios of *Blautia species* and increases levels of both colonic and blood IL-10 in mice, although a direct link between the two was not established [136]. Reductions in *Blautia* were found in children with type-I diabetes [137] and, among other changes, was associated with increased incidence of colorecal cancer in both humans [138] and mice [139]. However, high levels of *Blautia* were seen in human patients with inflammatory bowel disease [140], possibly revealing differences between human and mouse biology or perhaps representing a natural attempt to restore homeostasis. Reduction in *Eubacterium* is associated with increased incidence of Crohn’s disease [141] while increased presence is associated with both irritable bowel syndrome [142] and inflammatory bowel disease [143]. Meanwhile, *Clostridium coccodies* and *C. leptum* are protective against inflammatory bowel disease [92]. Furthering the complexity is the notion that any specific taxa altered by diet may represent a so-called “keystone species”, a species exerting greater effect than their numbers would suggest. As such, we cannot exclude any shift in the gut microflora as the etiology of immune alterations.

**Figure 2:**
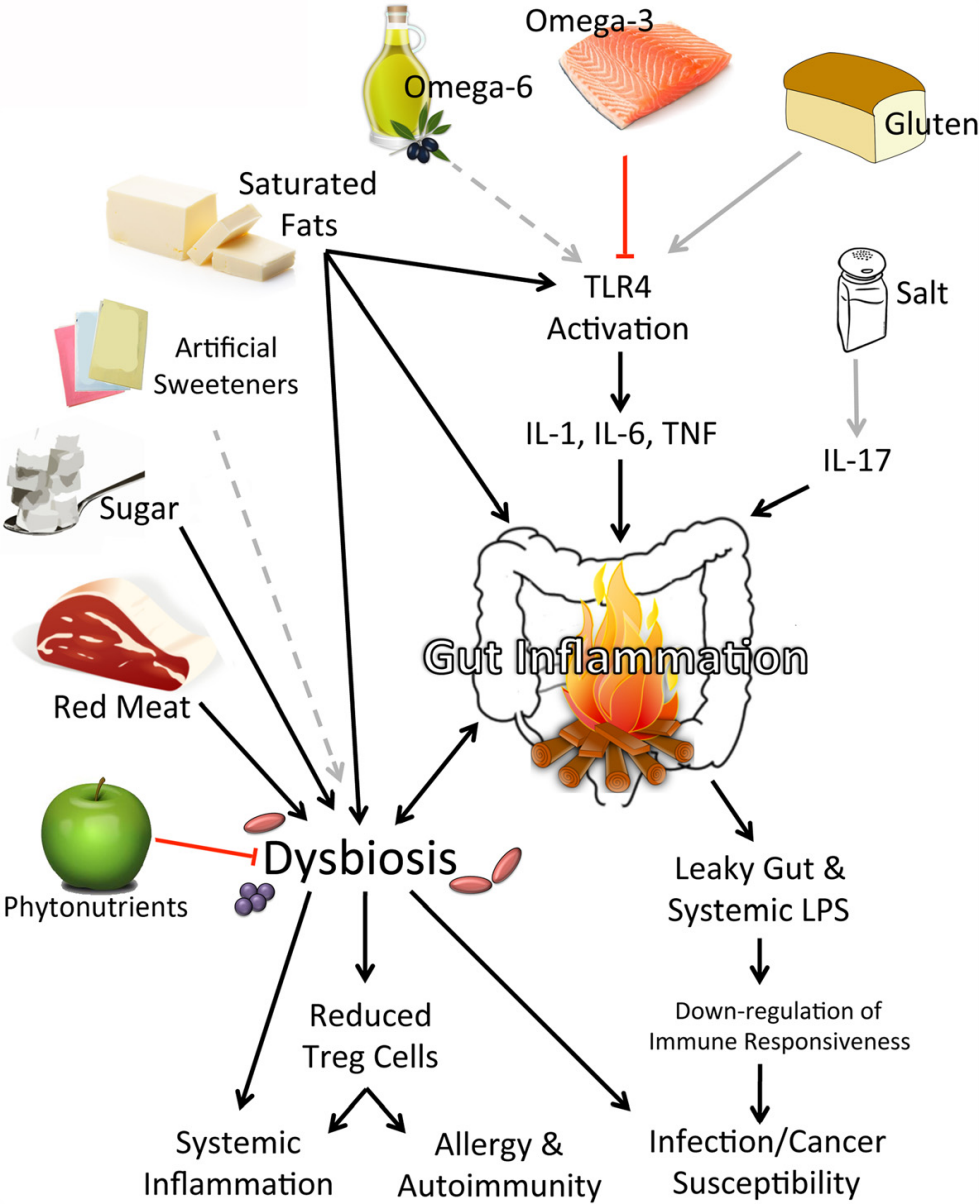
**Diagrammatic overview of the current mechanisms for macro-components of the modern diet altering susceptibility to infection, allergy, and autoimmunity.** Solid black lines indicate direct human evidence for enhancement is present; solid red lines indicate direct human evidence of inhibition exists; grey lines indicate only in vitro or animal model evidence exist currently; dotted lines indicate significant disagreement within the scientific literature. TLR4, Toll-like receptor 4; IL-, Interleukin; TNF, tumor necrosis factor; Treg, T-regulatory cell. All clip art and images sourced from free-for-us online repositories.

However, any hope for long-term benefits from probiotics may be limited by the need for dietary modification. Gordon and colleagues demonstrated that alterations in the mouse gut microbiome could prevent obesity, however these effects were dependent upon changing from a high-fat, low-fiber Western-style diet, to a healthier standard mouse diet [144]. While these findings are limited to mice, they raise a concern that taking probiotics may not be of benefit if the patient fails to eat a healthy diet. Additional, recent mouse studies [145] investigating how consumption of red meat may accelerate cardiovascular disease and inflammation in humans [146,147] suggest an additional and potentially serious limitation on probiotic supplementation. Dietary L-carnitine and choline, compounds abundant in red meat, are metabolized into trimethylamine-N-oxide (TMAO) by way of some normal gut commensals; in mice TMAO enhances atherosclerosis through disrupting cholesterol metabolism and foam-cell macrophage activity [145]. This may suggests that researchers cannot assume the safety of probiotic supplementation since bacterial species providing benefit to healthy individuals eating a healthy diet may hold the potential to become pathogenic when exposed to an unhealthy diet; however, like all studies limited to mice, human correlation will be needed. The benefits of dietary modification over supplementation is furthered by evidence showing that dietary supplementation does not increase longevity, indicating that probiotics and other commercial interventions such as tea or berry extracts are unlikely to counteract poor dietary habits [148]. Much work remains before the understanding of the effects of dysbiosis in humans reaches that of mice, however while definitive statements may be lacking, the preponderance of current evidence strongly suggests that the gut microbiome is a major contributor to human health and disease. The effects of macromolecules and immune function and dysbiosis are summarized in Figure 2.

### Immuno-nutrition in cancer

Although dietary factors are thought to account for up to one-third of cancers in Western nations [149], the complexity of immuno-nutrition is well highlighted in the research relating to cancer prevention. A typical meal may have thousands to bioactive compounds [150], distinguishing the effects of one from another is made all the more difficult by evidence that compounds may synergize or inhibit each other in respect to the development of neoplasms [151,152] as well as possible confounding by other environmental exposures such as smoking and infections (*H. pylori* for stomach [153], hepatitis B and C for liver [154], and Human-papilloma virus for cervical [154]). In general, chronic inflammation is associated with an increased risk of cancer [155], whether this is due to direct cellular damage, the previously discussed resultant down regulation of immune responsiveness, or a combination is unknown. There are associations between oral and esophageal cancers and high intake of alcohol, tobacco, and of scalding hot food or drinks [156]. Colon cancer risk appears to be worsened by high intake of red meat, salt-preserved meat, and fat, although the data is not completely conclusive [157-159]. Excessive intake of alcohol is associated with cirrhosis-induced liver cancer and is a risk factor for breast cancer [149,160]. Evidence on dietary risk factors for pancreatic, lung, prostate, and kidney are more controversial, but have been exquisitely reviewed [149].

Palmitic acid may potentiate iron-mediated toxicities and increase the rates of DNA mutations while inhibiting the normal apoptotic pathways [161-163]. Dietary intake of the saturated palmitic and steric fatty acids as well as the omega-9 oleic acid, may be independent risk factors for the development of colon cancer [164]. Simple sugars were thought to heighten cancer risk through several well-reviewed in vitro mechanisms [165], however more recent clinical analysis has not shown an increased risk of cancer attributable to sugar intake and suggests the original findings are more likely related to total caloric intake or glycemic load [166-168]. There is however, convincing evidence that obesity itself increases the risk of cancers of the breast, uterus, colon, esophagus, and kidney [169-171].

There is some evidence suggesting anti-tumor properties for vitamin E [172-175], vitamin D [176], and selenium among others [177]; the details of which have been well reviewed [150,178,179]. Meanwhile, initial excitement regarding pre-operative supplementation with omega-3 fatty acids improving outcomes in patients undergoing gastrointestinal cancer surgery [164,180] and for beta-carotine as a therapeutic in lung cancer has lessened after failures in double-blind follow up trails [181-183]. Overall, recent failed investigations into nutritional supplementation and cancer prevention may have weakened the enthusiasm for use of synthetic multivitamins in the prevention of cancer [182-185], perhaps indicating that the beneficial effects seen with increased natural consumption of these products [179,186-189] is due to bioactive compounds other than the measured vitamins or, more likely, that there are differences in how isolated synthetic molecules behave outside of the context of the intact food product. Not that use of supplements for other goals is unwarranted (such as iron for anemia or calcium and vitamin D for bone health) but for immune health, it appears whole dietary sources harbor the truly beneficial properties.

The exact mechanism of how any individual dietary element impacts cancer development is far from fully understood. Many of the reportedly protective vitamins and minerals share anti-oxidant properties, suggesting a mechanism more related to protection of DNA from damage than altered immune function [190-193]. Dietary modifications of the epigenetic methylation silencing of tumor suppressor and promoter genes have also been implicated [194,195]. Additionally, a number of association studies in humans and mice have linked the development of colon cancer to dysbiosis, particularly an increase in *S. bovis* and certain adhesive/invasive strains of *E. coli*[196-199]. Yet, while the role in, and mechanism for diet-induced immune dysfunction in cancer development cannot be outlined declaratively, given the association of neoplasms with certain dietary choices it seems unlikely that diets that worsen infection and/or induce chronic inflammation will be benign.

### Genetically modified (GM) foods

Another area of concern involves genetically modified organisms (GMO) in the food supply. While the debate around GM foods tends to be conducted in an all-are-good or all-are-bad format, some distinctions are notable in the literature. Potential benefits are highlighted by GM rice strains modified to produce high levels of beta-carotene. Vitamin A deficiency severely impairs immune function and thus any alleviation could produce dramatic benefit in parts of the world with both low nutrient supply and high exposure to pathogens [200]. While not without vocal detractors [201], use of GM rice is equivalent to supplement pills at providing adequate intake of vitamin A in children and thus offers a potentially life-saving benefit, as delivering beta-carotene through rice would be easier and more economically sustainable than through medication [202]. Whether sourcing vitamin A from GM crops suffers from the same shortcoming that pill-based supplementation have in regards to improving clinical outcomes [182] remains to be seen. Another potential benefit of GM technology, enhancement of crop yields, appears highly dependent on the specific modification in question and the area into which it is being deployed. Modifications that protect plants against select caterpillars or beetles greatly improve yield in places stricken with these pests, such as in parts of India [203], but make no measurable impact in places without infestations, such as the United States or Europe [204]. However, modification that impart drought-resistance to crops may hold promise for improving crop yields in parts of the United States, although their effects on human health are as yet untested [205]. Thus, modifications may protect against the harmful impacts of malnutrition, but only if they are correctly targeted [206].

The possibility of inducing allergic reactions through genetic modification was outlined when genetic elements from Brazil nuts were grafted into soy with deleterious consequences [207]. However, today GM food products must be screened for homology against all known allergens and future genetic modifications may even remove allergenic proteins from the common allergenic foods [208]. Yet, concerning evidence does exist against certain GM food practices. While many genetic modifications represent the grafting of one naturally occurring and routinely encountered gene into a commonly eaten food, others signify what can be thought of as “self-spraying” elements; some GM plants internally produce pesticides or pesticide inhibitors in a manner that holds little functional difference from externally spraying the compound – examples include Bacillus thuringiensis (Bt) producing and glyphosate-resistance plants. Since pesticide-resistance genes tend to encourage increased use of pesticides [204], it is a concerning finding that pesticides like glyphosate induces cellular death in human umbilical, placental, embryonic, and peripheral blood mononuclear cells at physiologic levels [209-211]; further studies will be needed to either confirm or alleviate these concerns. In animal models, the combination of pesticide-producing GM maize and pesticide-resistant GM soy led to increased rates of severe stomach inflammation [212], although GM maize alone did not have significant effects on either inflammation or the make up of the gut microbiome of pigs [213] and direct consumption of high doses of Bt insecticide did not induce acute toxicity in humans or toxicity in mice [214].

However, an additional concern was raised when studies revealed that functional genes, from both industrial and natural sources, ingested by animals could be internalized by gut bacteria, these bacteria transcribe the engrafted genes into functional proteins, and the genetic changes could be inherited by offspring via microbiome transfer [215-218]. Human corollary was uncovered when researchers showed that an intact and functional industrial gene could be found in bacteria from the small bowel of patients with ileostomies [219]. Research subjects with intact gastrointestinal tracts did not show evidence of the gene surviving the large bowel. While the authors concluded that such modifications were safe because only a small number of small-bowel bacteria expressed the functional pesticide-resistance gene, the hypothetical potential for transferring the production of harmful compounds to the microbiome in a manner that would circumvent gastric-acid inactivation was shown. In theory, the ability to transfer genetics to the gut microbiome could be utilized for therapeutic purposes, but would be limited by the need to assure consistent and non-toxic levels. Yet while the lack of direct investigation into the human health impacts of GM foods preclude definitive comment to either confirm or alleviate the concern for harm, research on industrial GM crops are subject to patent-law limitations, meaning that any report of findings must have the manufacturer’s approval prior to publication [220]. Thus the potential for conflict of interest and suppression of evidence exists and paradoxically may fuel some of the scientific ignorance and mistrust driving the all-or-none debate.

### Immunity driving nutrition

The original name for TNF-alpha was cachectin, derived from its observed ability to induce profound weight loss (cachexia) and appetite suppression [221]. Although the exact pathology is complex, patients with chronic infections could find their appetite chemically blocked, opening the possibility for further worsening of immune function and infection from poor nutrient intake. Loss of appetite and depression from some pro-inflammatory cytokines [2] may limit palatable food options in ways that increase the risk for nutrient deficiencies. Pro-inflammatory cytokines also affect the glucocorticoid pathway, with implications on cortisol physiology and thus on stress, metabolism, and development [221].

Beyond the well-known common antigens, coloring and flavoring additives such as annatto and carmine may cause anaphylaxis in sensitized patients and drive labeling laws that influence dietary choices [222]. Unfortunately, earlier recommendations for allergen avoidance during pregnancy, breast feeding, and the child’s early years may have done little to decrease allergic disease burden; while definitive conclusions cannot yet be drawn, current evidence does not support allergen avoidance during gestation or nursing, and preliminary results from ongoing studies have suggested early introduction of foods may prove to be protective [223,224]. Allergic disease may subsequently drive limitation in nutritional choices such as in children with atopic dermatitis whom tend to avoid nuts, dairy, egg, fish, wheat, and/or artificial colorings [225]. While every child should be evaluated for nutritional deficiencies on an individual basis, children on allergy-driven elimination diets have an increased risk for calcium and omega-3 deficiency [225,226]. Therefore, clinicians should be attuned to both the emotional and immunologic impacts of chronic inflammation and immune dysfunction in their patients.

### Economic considerations

Financial limitations are often cited as a primary factor behind the higher consumption of unhealthy food products in a nation’s poor [227,228]. Yet, rising medical costs stemming from diet-related illness have extended these economic limitations into middle-income families [229] and are thus capable of generating a downward spiral of poor diet begetting medical illness, illness increasing poverty, and poverty further entrenching a poor diet. However, burdensome out-of-pocket medical costs [230] may be forcing our society to realize that no matter how much cheaper a pro-inflammatory meal may be at the register, the cost savings will be quickly erased by any resultant medical illness. Costs of obesity not shouldered by the patient are also mounting, one 2010 study asserts that obese Americans will average additional annual medical cost on the order of $1,152 for men and $3,613 for women when you factor in loss of productivity; subjects that were significantly underweight also saw increased medial bills [231]. Irrespective of whether such expenses are carried by a system that is fully socialized, privatized, employer-based, or any combination therein, dietary choices will have large-scale impacts on the economy. While poverty will continue to limit dietary options, some fast food chains are improving the health of their menus [232], for example where there was once only soda and fries as fast food side items, many chains now offer options for apples and milk. This trend will hopefully continue and perhaps even spread to the sadly lagging convenience stores and, in particular, the processed food manufacturers [232,233] if the public furthers the realization that cheap food is not inexpensive if it is also unhealthy.

### Caveats

While the plentiful bounty of the Western world may protect us from the harmful immune effects of micro- and macronutrient deficiencies, the over abundance of many substances simultaneously enhance inflammation while muting our immune system’s ability to respond to and ultimately control infections. The nutritional elements discussed herein are certainly not the entire explanation for the disease patterns seen in industrialized societies, and additional immune harms stemming from our increasingly sedentary lifestyles, altered infectious exposures, as well as increased exposure to pollutants warrant their own reviews [2,234,235]. We should note that the Western diet’s additional, more established, propensity for damaging metabolic homeostasis also causes *secondary* immune dysfunction through resultant diseases such as diabetes, adding greater emphasis on eating a healthy diet; the impacts of diet on the risk of metabolic disorders and the immune defects in diabetes have also been well reviewed [236-240]. Additionally, although less established, the impacts of exercise on immunity and overall health are worthy of separate examination [241,242]. One final caveat of note is that many of the studies cited employ isolated nutrients for testing, and thus an interesting area for further research will be the difference between consumption of these items in their naturally diverse combinations as compared to the homogenized forms found in processed foods or supplements; peanuts, for example, may contain over eight different fatty acids [243], fruits have varying ratios of simple and complex carbohydrates, whereas many convenience foods contain a predominance of oleic and palmitic saturated fatty acid, omega-6 fats, and fructose syrups [1,244-246]. In effect, we must investigate the difference between teaching the body to harvest fat, carbohydrates, and protein from a proverbial fish rather than giving the body these pre-extracted nutrients.

## Conclusions

Table 1 serves as a summary outline of the data on immuno-nutrition focused upon in this review. In summary, there is enough quality, direct human evidence to conclude that many of the dietary choices in today’s modern society appear to have harmful impacts on our immune system and likely on the immune system of our offspring; while many of the remaining conclusions related to the modern diet’s deleterious influence can only be extrapolated from in vitro and/or animal models, given the sheer volume of evidence, predictions of similar human harm seem far from unreasonable (Table 1). Although promise remains, it also appears unlikely that synthetic supplements or probiotics will be able to fully counterbalance the damage of our dietary choices, let alone undo them, if they are not accompanied by lifestyle modifications. Of potentially greatest concern, our poor dietary behaviors are encoded into both our DNA scaffolding and gut microbiome, and thus these harmful immune modifications are passed to our offspring during their most critical developmental window. Therefore, given the scope of influence, the vast economic impacts, and the potential for trans-generational inheritance, the dietary impacts on immune health should thus, at minimum, be afforded a level of attention equal to that given to the dietary impacts on cardiovascular health.

**Table 1:** Summary of the immune impacts of dietary components and the nutritional impacts of various disease states

**Macronutrient**	**Immunologic impact**	**In vitro evidence**	**Animal models**	**Human evidence**	**Reviews**
Simple sugars	- Reduced phagocytosis	24	—	**25**, *94*–*95*, *100*, **101**, 102	92, 103, 104
	- Increased inflammatory cytokines production				
	- Dysbiosis				
Complex sugars	- Reduced inflammatory cytokine production	27	33	**27**, *28*, 29–30, *31*	26, 32
	- As part of intact food substance, may reduce risk of certain diseases				
	- Reduced dysbiosis				
Artificial sweeteners	- Mostly unknown or unproven	36-37, 104-107	35, 40	*34*	—
	- Potential contributor to inflammatory bowel disease				
	- Stevioside may enhance phagocytosis and T/B-cell mitogen responses				
Salt	- May increase IL-17 and worsen autoimmune disorders	—	41-42	—	—
Saturated fat	- Alterations in prostaglandin pathway and antioxidant mechanisms	45, 47–49, 59, 61–62, 161-163	50-52, 55, 60	**54**, **56**, *57*–*58*, *164*	43-44, 53, 63
	- TLR2, and TLR4 activation; CD14 alterations				
	- Increase gut inflammation and reduce gut barrier function				
	- Worse outcomes in sepsis; Increased risk of autoimmunity, allergy, certain neoplasms				
	- Dysbiosis				
Trans fat	- Mostly unknown	—	—	—	64
	- Increased IL-6 and CRP levels				
Omega-6 fatty acids	- Increased inflammation via TLR4 activation	67	52, 66	**65, 68**	53, 64
	- Dysbiosis				
Omega-3 fatty acids	- Reduced inflammatory cytokines and transcription factors	48, 74	80-82, 136	*75-77*	63, 72–73, 79
	- Increased resolvin and protecin production				
	- Increased IL-10				
Gluten	- Possible TLR4 activation; studies limited to animal models	83	83	84-87	88-89
	- Induction of Celiac symptoms in patients with HLA-DQ2 or HLA-DQ8				
Red meat	- Mostly unproven; studies limited to animal models	—	145	142, *147*	64, 149
	- Increased endothelial inflammatory, activation of foam-cell macrophages				
Genetic modification	- Mostly unknown	209-211	212-218	**202**, *207*, **219**	—
	- Reduction vitamin A or calorie deficiency depending on modification/location of deployment				
	- No apparent impact on allergic disease				
	- Increased exposure to pesticides				
	- Potential for transmission of functional genes into small bowel bacteria				
**Pathologic disorder**	**Effect on nutrition and/or immunity**	**In vitro evidence**	**Animal models**	**Human evidence**	**Reviews**
Obesity	- Increased inflammatory cytokines, immunologic tolerance to inflammatory cytokines	12	19	11, *13*–*15*, 16–18, *21*, *169-171*	7, 20, 149
	- Reduced leukocyte numbers and function				
	- Reduced control of infection, heightened rates of certain neoplasms				
	- Overproduction and eventual tolerance of leptin				
	- Dysbiosis				
Anorexia and bulimia	- Reduced monocyte, neutrophil, and T-cell numbers and function	—	—	*22*	23
	- Reduced complement function				
	- Any disorders related to micronutrient disorders				
Dysbiosis	- Maternal transmission leading to immune alterations in the offspring	47, 96–98, 104–107, 198-199	52, 93, 111, 139, 144, 196	*94-95*, *100*, **101**, 102, 109, *110*, **116**, **131**–**135**, 138, 141–143, *197*	91-92, 99, 103
	- Epigenetic changes altering offspring immunity via paternal inheritance				
	- Reduced regulatory T cell numbers				
	- Worse outcomes in sepsis; Increased risk of autoimmunity and allergy				
	- May increase likelihood of obesity				
	- May increase risk of certain neoplasms				
Chronic inflammation	- Reduced appetite and weight loss	—	—	**155**	2
	- May increase risk of certain neoplasms				
Food allergy	- Avoidance diets predisposing to deficiency in calcium and omega-3	—	—	222, **223**, *224*, 226	—

Citations are organized by the primary models used in the research, cell culture (In vitro), animal, or direct human effects. For studies involving human data: further notation indicates cross-sectional studies (standard font); longitudinal study designs both prospective or retrospective or reviews discussing longitudinal evidence (italic font); or interventional trials or reviews discussing intervention studies (bold font). The citations provided are not meant to be all-inclusive and thus additional cited reviews of note are also provided; no additional annotation is provided in the review article column.

## Competing interests

The author declares that he has no competing interests.
